# Biases and Power for Groups Comparison on Subjective Health Measurements

**DOI:** 10.1371/journal.pone.0044695

**Published:** 2012-10-24

**Authors:** Jean-François Hamel, Jean-Benoit Hardouin, Tanguy Le Neel, Gildas Kubis, Yves Roquelaure, Véronique Sébille

**Affiliations:** 1 EA 4275: Biostatistics, Clinical Research and Subjective Measurements in Health Sciences, Faculty of Pharmaceutical Sciences, University of Nantes, Nantes, France; 2 Methodology and Biostatistics Unit, University of Angers, Angers, France; 3 Laboratory of Ergonomics and Epidemiology in Health at Work, University of Angers, Angers, France; Cardiff University, United Kingdom

## Abstract

Subjective health measurements are increasingly used in clinical research, particularly for patient groups comparisons. Two main types of analytical strategies can be used for such data: so-called classical test theory (CTT), relying on observed scores and models coming from Item Response Theory (IRT) relying on a response model relating the items responses to a latent parameter, often called latent trait. Whether IRT or CTT would be the most appropriate method to compare two independent groups of patients on a patient reported outcomes measurement remains unknown and was investigated using simulations. For CTT-based analyses, groups comparison was performed using t-test on the scores. For IRT-based analyses, several methods were compared, according to whether the Rasch model was considered with random effects or with fixed effects, and the group effect was included as a covariate or not. Individual latent traits values were estimated using either a deterministic method or by stochastic approaches. Latent traits were then compared with a t-test. Finally, a two-steps method was performed to compare the latent trait distributions, and a Wald test was performed to test the group effect in the Rasch model including group covariates. The only unbiased IRT-based method was the group covariate Wald’s test, performed on the random effects Rasch model. This model displayed the highest observed power, which was similar to the power using the score t-test. These results need to be extended to the case frequently encountered in practice where data are missing and possibly informative.

## Introduction

Subjective health measurements are increasingly used in clinical studies to assess patients’ perception of their own health [1], [2]. For example, they allow assessing phenomena such as quality of life, tiredness, depression or anxiety. These phenomena are called latent variables because they can not be directly observed nor measured. However, their effects can be accessible through the analysis of other variables that are directly observable.

Assessing these subjective measurements is usually done by using self-assessment questionnaires called patient reported outcomes (PRO) which consist of a set of questions often called items. Two strategies have been developed to analyse such questionnaires: the Classical Test Theory (CTT) and the Item Response Theory (IRT). These theories provide different conceptual frameworks for the analysis of PRO, each being based on several hypotheses that have to be tested before analysis. CTT is based on the assumption of a linear model explaining the individual observed score by a theoretical individual score plus a stochastic error term. Such an hypothesis can be tested using Cronbach’s alpha [3]. On the other hand, IRT is based on the assumption of a logit model explaining the individual item responses by a latent parameter, often called latent trait. Such an hypothesis can be tested using R1m global tests of item fit [4].

With CTT, the item responses are combined to provide scores allowing analysing the data. In most cases, these scores should be considered as ordinal qualitative measurements of the latent variables studied, and thus cannot be considered as interval measurements [5], [6]. It means that a unit difference characterizes the same amount when measured from different initial levels on the latent trait scale.Therefore, a given score variation cannot be associated with a given latent variable variation and one should not rely on CTT to quantify an expected effect or a clinical significance threshold [7], [8].

With IRT, the latent variable is quantified by measuring the latent trait. The latent trait, estimated by modelling the probability of an observed response to an item, can always be considered as a quantitative variable with interval measurement properties [9]. Then, the IRT systematically allows both quantifying an expected effect or the clinical relevance of an observed difference, but also highlighting latent trait differences between compared groups.

A simple and widely used IRT model, adapted to the analysis of dichotomous items, is the Rasch model [9]. In this model, the probability of a specific response (e.g. positive or negative answer) is modelled as a function of person and item parameters. Person parameters pertain to the latent trait level of people who are evaluated while item parameters pertain to the difficulty of the items (in a Rasch model, the difficulty of an item is equal to the latent trait of an individual who would have an equal probability of responding positively or negatively to this item). Person parameters can then be interpreted as a propensity to respond positively to each item.

This model can be grasped in different ways: all the individual latent traits can be considered as a set of fixed effects (this is known as the fixed effects Rasch model), or as realizations of a random variable assumed to be normally distributed (this is known as the random effects Rasch model). With a fixed effects Rasch model, the purpose is to assess for each individual the value of his/her individual latent trait. On the contrary, with a random effects Rasch model, the purpose is to directly estimate the parameters of the overall distribution of the latent trait: in the case of a normal distribution, two parameters are estimated: the mean and the variance of the latent trait. Finally, if the sample consists of individuals coming from potentially distinct populations, it is possible to add a group covariate in the random effect model.

Several methodologies can be used to compare two samples of patients on PRO data coming from an IRT-based or a CTT-based validated questionnaire. These methodologies depend on the use of CTT or IRT, and on the chosen model to estimate latent traits if IRT is used. Whether one approach would be more suitable than another is still under debate and not perfectly known to date.

The aim of our study is to evaluate and to compare different group-comparison methods from IRT-based and CTT-based models. The statistical properties of the different methods either based on CTT or IRT were assessed and compared by simulations regarding the type I error, power, and bias in parameter estimates.

## Methods

### Simulation Study

One of the most relevant strategies to explore the empirical properties of comparison methodologies is to perform them in perfectly known contexts. Then, the “true” statistical conclusion is known, and can be compared with the observed conclusion. For example, to study the type I error of a group comparison test, it should be performed on two samples both drawn from the same population. The proportion of rejections of the null hypothesis should actually correspond to the probability of finding a difference that does not exist in reality. In contrast, this test should be performed on two samples drawn from different populations to study its power.

An appropriate strategy to know *a priori* the origin of the analysed samples is to generate those using Monte-Carlo simulations. Unlike a real data study, data resulting from Monte-Carlo simulations should allow differentiating whether a statistically significant difference is linked to a real difference or to the first order risk of the considered test.

In our study, we generated the data using Monte Carlo simulations with a Rasch model. Doing so allowed us to assume that the simulated questionnaires had been previously validated to be analysed either with a Rasch model or with CTT: the assumptions needed to analyse a data with a CTT-based model were necessarily fulfilled through a data satisfying the assumptions of a Rasch model [10].

Several parameters combinations were considered to generate the simulated data.

- For each simulation, we simulated two samples *A* and *B* of equal size 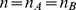. The sample size per group ranged from 50 to 400 subjects to reflect sample sizes commonly encountered in clinical research studies.
- The latent trait distribution was defined as normal. The normal distribution was chosen to respect the hypothesis related to the implementation of a random effects Rasch model.
- The latent trait distribution variances were equal to 1 to be within the framework of reduced data, and so to overcome the problem of the measurement scale. Thus, the differences in latent trait and in difficulties were only expressed in terms of standard deviation fraction.
- The simulated differences between the means of latent traits 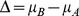 were set at 0, 0.2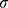, 0.5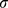 and 0.8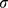. The latent traits mean for groups *A* and *B* were therefore respectively equal to 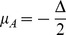 and to 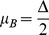. A difference set at 0 corresponded to a lack of effect, and allowed estimating the tests type I error by computing the proportion of rejection of the null hypothesis. A difference set at 0.2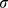, 0.5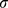 or 0.8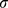 corresponded respectively to a small, medium or large effect size [11] and allowed estimating power by computing the proportion of rejection of the null hypothesis.
- The items were defined as dichotomous, so they could be analysed by a Rasch model. Each positive response was coded as 1 and each negative response as 0. The number of items was set at 5 or 10 in accordance with the size of the subscales of the most commonly used questionnaires to measure PRO. For example, the NHP consists of 6 subscales composed of 3 to 9 dichotomous items [12]. As well, the SF-36 consists of 8 subscales composed of 2 to 10 items, 2 subscales being only composed of dichotomous items (Emotional Role Limitation, and Physical Role Limitation), the others of polytomous items [13].
- These items difficulties were defined as the percentiles of a standard normal distribution or as the percentiles of an equiprobable mixture of two Gaussian distributions. These two possibilities allowed considering two different situations that can be encountered in practice. The normal distribution reflected the situation where the questionnaire was perfectly adapted to a population with normally distributed latent traits. Evenly distributed items difficulties allowed considering the score as an interval measurement. The bimodal mixture corresponded to a more irregular and probably more realistic items difficulties distribution. Gaussian parameters of this mixture were then chosen to distinguish two groups of items within the scale: a first group of items whose difficulty values were very close, and a second whose difficulty values were more far apart. Such a distribution involved a poorer match to the latent trait distribution and thus floor or ceiling effects, and did not allow considering the score as an interval measurement.
- The individual items responses were generated by Bernoulli trials, after calculating for each individual the probability of response to each item by a Rasch model.
- Each parameter combination of the simulations was replicated 1000 times.

The details of the chosen simulation parameters are presented in table 1.

**Table 1:** Possible values of the different simulation parameters.

Parameters	Values
Sample size 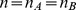	50100200300400
Latent trait distribution: 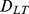	Standardized normal distribution
Differences between the latent traits means: 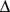	0σ 0.2σ 0.5σ 0.8σ
Mean of the latent traits	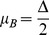 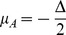
Variance of the latent traits	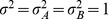
Number of items: 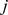	5 10
Items difficulties distribution: 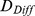	• Standardized normal distribution
	• Equiprobable mixture of two gaussian
	distributions with parameters 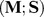
	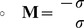
	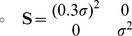

### Statistical Analysis

For each simulation of each parameters combination, the individual scores 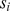 for person 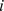 (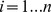) were defined as the sum of the items positive responses. The latent trait analysis (IRT) has been performed with fixed effects and random effects Rasch models. These analyses were conducted assuming three distinct cases:

- One could consider the difficulty parameters as unknown, which required to estimate them during the IRT analysis,
- One could assume these parameters as already known (eg estimated during previous studies, or coming from items banks such as the quality of life item bank PROMIS [14]). In this case, they were not estimated during the analysis. Knowledge of these parameters was then envisaged in two ways:
  The difficulty parameters were considered as well known: the fixed values of the difficulty parameters used during the analysis 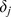 were equal to the simulated difficulties 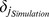

  The difficulty parameters were considered as imperfectly known, or known with error: the fixed difficulty parameters values used during the analysis 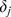 were randomly drawn from uniform distributions U(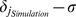; 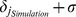)

### The Rasch Model

One of the commonly used IRT model adapted to the analysis of dichotomous items is the Rasch model [9]. Let 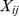 be the dichotomous variable representing the response of person 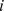 (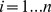) to an item 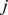 (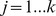). For a questionnaire containing 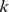 dichotomous items, the model can be written as follows (eq.1):
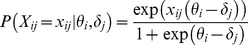
(1)where 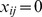 for a negative response and 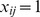 for a positive response, 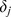 is the difficulty associated with item 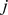, and 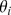 is the individual value of the latent trait for patient 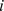.

When all the individual latent traits are considered as a set of fixed effects, the Rasch model is known as a fixed effects Rasch model, while when the individual latent traits are considered as realizations of a random variable assumed to be normally distributed, the Rasch model is known as a random effects Rasch model.

### The Fixed Effects Rasch Model

The estimates of the fixed effects Rasch model parameters were obtained using a two-step procedure, providing consistent estimators [15]–[17]. The estimates of the items difficulty parameters were obtained with conditional maximum likelihood, given the individual scores 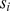 (eq.2). The estimates of the individual latent traits were then obtained with weighted maximum likelihood (WML) (eq.3). This entire procedure is known as the CML procedure. By extension, in this study, a fixed effects Rasch model will be called CML-model.

Let 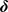 be the *k*-vector of items difficulty parameters 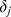, 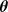 be the *n*-vector of individual latent-traits, 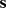 be the *n*-vector of individual scores 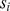, 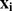 be the *k*-vector of the items responses for the 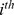 individual and 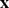 be the 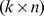-vector of the items responses for all the *n* individuals.

The 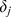 parameters are consistently estimated by maximizing the conditional likelihood (eq.2):
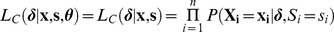
(2)where 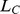 is the conditional likelihood given the subject’ s scores 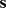.

The 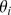 parameters are then estimated without biases by maximizing the weighted likelihood 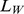 (eq.3):
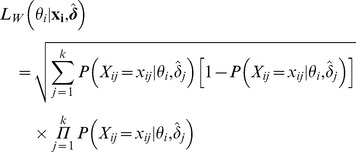
(3)As with any maximum likelihood estimating procedure, the parameters estimated with the CML procedure are asymptotically normally distributed according to a normal distribution with mean equal to their maximum likelihood estimator. To assign to each individual his own latent trait value, we must define a decision rule based on this estimated distribution. It will be defined in section: *“Different possible estimates of the individual latent traits”*.

### The Random Effects Rasch Model

The estimate of the random effects Rasch model parameters were obtained with marginal maximum likelihood (eq.4), known as the MML procedure [16]. The latent trait was then considered normally distributed with mean 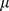 and variance 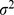. By extension, a random effects Rasch model will be called MML-model in this study.

The 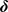, 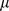, and 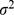 parameters can be consistently estimated by maximizing the marginal likelihood 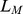 (eq.4):
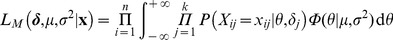
(4)where 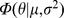 is the cumulative distribution function of the studied population latent trait 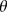, assumed to follow a normal distribution with parameters (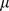; 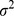).

The estimators of each individual latent trait, assumed to be normally distributed, could be obtained afterwards, with expected a posteriori Bayesian (EAP) estimates [17], [18]. EAP estimates are obtained by taking the expectation of the posterior density function of 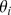, conditional on 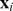 and 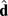 (eqs.5 & 6).
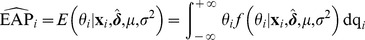
(5)

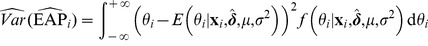
(6)where 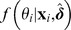 is the posterior density function of 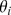, conditional on 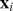 and 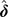.

### Including a Group Effect in a Rasch Model

The group effect can be represented by a covariate in the formulation of the Rasch model [19]. The individual latent traits 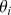 are then decomposed into a part related to the group (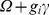), and a part related to the individual (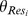). The model is then written as (eq.7):
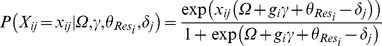
(7)where 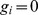 if the 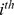 individual is in the first group and 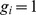 if the 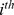 individual is in the second group. The average latent trait in the first group is equal to 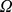, and in the second group equal to 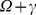. The individual latent traits 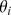 can then be computed as: 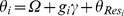.

We did not perform any fixed effects Rasch model with group covariates. Such a model would be unidentifiable, estimates for the Rasch model with fixed effects being computed conditionally on the individuals. It was only possible to include a group covariate within a random effects Rasch model. This model has been called MML-Cov.

### Different Possible Estimates of the Individual Latent Traits

Two different ways of estimating the individual latent traits can be proposed.

- The most intuitive choice for an individual latent trait value estimate performed with a CML, MML or MML-Cov model, is probably the estimated mean of the individual latent trait distribution. For the CML model, these are the WML-CML estimates, and for the MML and MML-Cov models, these are the EAP-MML and EAP-MML-Cov estimates. (EAP-MML-Cov is then computed as the sum of 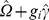 and 
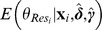
)
- As we cannot know the true values of individual latent traits, but only their distributions, the individual latent traits values can be defined as plausible values (PV) coming from these distributions [20], [21]. The latent trait of each individual is then assigned from a draw from its estimated latent trait distribution. For the CML model, these are the PV-CML estimates, for the MML model the PV-MML estimates, and for the MML-Cov model, the PV-MML-Cov estimates.

### Different Methods to Compare Two Groups on PRO

Different methodologies have been proposed for comparing two groups of subjects 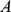 and 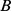 on PRO data.

- When using CTT, the groups are compared with a t-test using mean scores. In our study, this method has been called score t-test.
- When using IRT, groups can be compared using several tests.

Individual latent traits values can be compared with a t-test, whether these are defined as the estimated means of the individual latent traits distributions (WML-CML, EAP-MML and EAP-MML-Cov methodologies) or as plausible values coming from these distributions (PV-CML, PV-MML and PV-MML-Cov methodologies). For example, this is how the most currently used software for Rasch analysis: RUMM software [22] compares individuals groups: the individual latent traits, estimated using WML-CML methodology, are compared with a t-test.
Using the MML-Cov model, it is possible to perform a group comparison by testing the nullity of the parameter associated with the group covariate with a Wald test. In our study, this method has been called “Wald-test”.
Mislevy [23] noted that obtaining the variance estimate of a the latent traits within a group by calculating the variance of their individual estimates is biased because it only corresponds to the between-individual variance estimate, regardless of the within-individual variance estimate [24], [25]. With multiple imputations of plausible values (MI method), it is possible to estimate the distribution parameters of the latent traits of each group, taking into account both the between-individual and the within-individual variance. One can then compare the groups with a t-test. In our study, these methods have been called IM-CML, IM-MML or IM-MML-Cov according to the model used (CML, MML or MML-Cov model).

This methodology was developed for large scale surveys used in educational sciences (eg the PISA, TIMSS and NAEP studies). the number of imputations used was then between 3 and 5. Rubin recommends making between 2 and 10 imputations [24]. In our study, we performed five imputations to be comparable to studies using this methodology.

Finally, it was proposed to perform groups comparisons with a two-step procedure (this procedure is called 2-Steps method [26]). The first step is to estimate the difficulty parameters with MML method, and the second one is to separately estimate the latent traits distributions parameters for each group by performing a random effect Rasch model in each of these groups, with difficulty parameters set to the estimated values obtained during the first step. Since it is possible to estimate with this method the mean and the variance of the latent traits for each group, it is then possible to compare the groups by performing a t-test.

**Figure 1:**
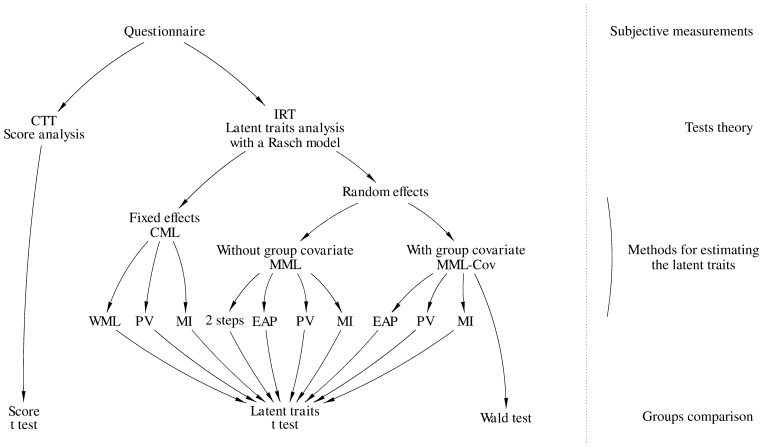
Different methods to compare two groups of patients on subjective measurements. CTT: Classical Test Theory, IRT: Item Response Theory, CML: conditional maximum likelihood, MML: marginal maximum likelihood, MML-Cov: MML with group covariate, WML: weighted maximum likelihood, EAP: expected a posteriori, PV: plausible values, MI: multiple imputations of PV.

All these methodologies are summarized in figure 1. All the tests were performed with a threshold 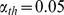.

### Comparison of Methods

To compare the methods to analyse PRO data, four criteria were studied: the type I error, the power, the position bias and the dispersion bias.

- The type I error was classically obtained by calculating the proportion of rejection of the null hypothesis among the 1000 replications of the same parameters combination when 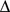 was set to 0. A test of equality between the observed type I error and 0.05 was then performed with a t-test.
- The power 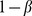 was obtained by calculating the proportion of rejection of the null hypothesis among the 1000 replications of the same parameters combination when 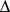 was different from 0. It was considered that a power variation of less than 0.05 was not relevant in practice.
- When the methodology was based on IRT:
  We estimated the difference between the latent traits means of each group by computing the average of the differences between the means of the latent traits of the groups 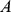 and 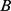 over the 1000 replicated simulations: 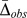. This average was then compared to the simulated difference 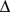 with a t-test. When 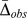 was significantly different from 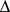, we concluded to a statistically significant position bias. It was then considered that a position bias of less than 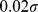 when 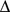 was equal to 0, or less than 10% of 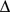 when 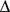 was different from 0 was not relevant in practice.
  We assumed that the variances of the two groups were equal: 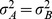. We estimated the latent traits variance of each group by computing the average of the latent traits variances over the 1000 replicated simulations: 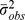. This average was then compared to the simulated common variance 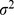 with a t-test. When 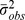 was significantly different from 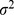, we concluded to a statistically significant dispersion bias. It was then considered that a bias of less than 10% of 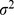 was not relevant in practice.
- When the methodology was based on CTT:
  We estimated the difference between the score means of each group by computing the average of the differences between the means of the scores of the groups 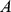 and 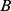 over the 1000 replicated simulations: 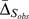. This average was then compared to the true value of group effect 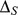 with a t-test. When 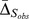 was significantly different from 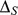, we concluded to a statistically significant position bias. It was then considered that a position bias of less than 10% of 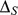 was not relevant in practice.

The true value of group effect 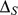 was not known and was approached using the difference of the expected score in each group.
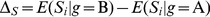
(8)


The expected score in each group was computed as follows:
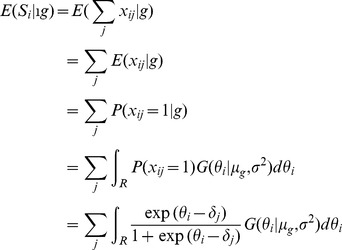
(9)with 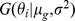 the normal distribution with mean 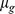 and variance 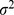. These integrals can be estimated using Gauss-Hermite quadratures

  We did not estimate the dispersion bias when the methodology was based on CTT.

Simulations and statistical analyses were performed with the Stata 11.0 software and the Gllamm package [27].

## Results

### Type I Error

The type I error level was similar whether the item difficulties were considered unknown, well known or imperfectly known. We will only present the observed type I errors for unknown difficulties that had to be estimated (table 2).

**Table 2:** Type I errors of the different methodologies for comparing groups on subjective measurements, for different simulation parameters; the difficulties are considered unknown.

				CTT	IRT										
					CML			MML				MML-Cov			
*D_Diff_*	*j*	*n*	Δ	t-test	WML	PV	MI	EAP	PV	MI	2 steps	EAP	PV	MI	Wald
Norm.	5	50	0	**0.036**	0.040	0.049	**0.001**	**0.037**	0.041	**0.000**	**0.173**	**0.315**	**0.212**	**0.071**	0.042
		200	0	0.058	0.052	0.048	**0.005**	0.058	0.046	**0.003**	**0.158**	**0.338**	**0.210**	**0.089**	0.058
		400	0	0.046	0.053	0.049	**0.001**	0.047	0.039	**0.001**	**0.172**	**0.342**	**0.216**	**0.082**	0.047
	10	50	0	0.048	0.051	0.054	**0.008**	0.047	0.042	**0.004**	**0.126**	**0.211**	**0.147**	0.060	0.054
		200	0	0.042	0.050	0.050	**0.004**	0.045	**0.038**	**0.002**	**0.109**	**0.203**	**0.158**	0.059	0.044
		400	0	0.051	0.045	0.052	**0.007**	0.050	0.045	**0.007**	**0.117**	**0.210**	**0.136**	0.059	0.050
Mixt.	5	50	0	0.042	0.044	0.052	**0.002**	0.042	0.039	**0.000**	**0.181**	**0.376**	**0.222**	**0.084**	0.044
		200	0	0.050	0.049	0.043	**0.004**	0.050	0.042	**0.001**	**0.172**	**0.336**	**0.198**	**0.082**	0.050
		400	0	0.050	0.052	0.045	**0.004**	0.050	**0.030**	**0.003**	**0.163**	**0.338**	**0.181**	**0.089**	0.051
	10	50	0	0.051	0.052	0.048	**0.010**	0.054	0.041	**0.005**	**0.136**	**0.228**	**0.168**	0.057	0.057
		200	0	0.048	0.045	0.040	**0.005**	0.050	**0.032**	**0.005**	**0.105**	**0.199**	**0.167**	0.056	0.052
		400	0	0.048	0.045	0.046	**0.011**	0.047	0.042	**0.007**	**0.113**	**0.201**	**0.143**	0.057	0.048

CTT: Classical Test Theory, IRT: Item Response Theory, CML: conditional maximum likelihood, MML: marginal maximum likelihood, MML-Cov: MML with group covariate, 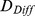: items difficulties distribution, 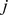: number of items, 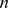: sample size, 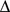: difference between the latent traits means, WML: weighted maximum likelihood, EAP: expected a posteriori, PV: plausible values, MI: multiple imputations of PV, Norm: normal distribution, Mixt: equiprobable mixture of two normal distributions.

Estimated type I errors significantly different from 0.05 appear in bold.

The type I errors observed for the score t-test, WML-CML, PV-CML, EAP-MML, PV-MML and Wald-test methods were not significantly different from 0.05. MI-CML and MI-MML methodologies minimized the type I error, while EAP-MML-Cov, PV-MML-Cov, MI-MML-Cov and *2 Steps* methodologies increased the type I error, whatever the values of the simulation parameters.

### Power

The methods for which the observed type I errors were significantly greater than 0.05 were excluded from the power analysis. We therefore excluded EAP–MML-Cov, PV–MML-Cov, MI–MML-Cov and *2 Steps* methods.

**Figure 2:**
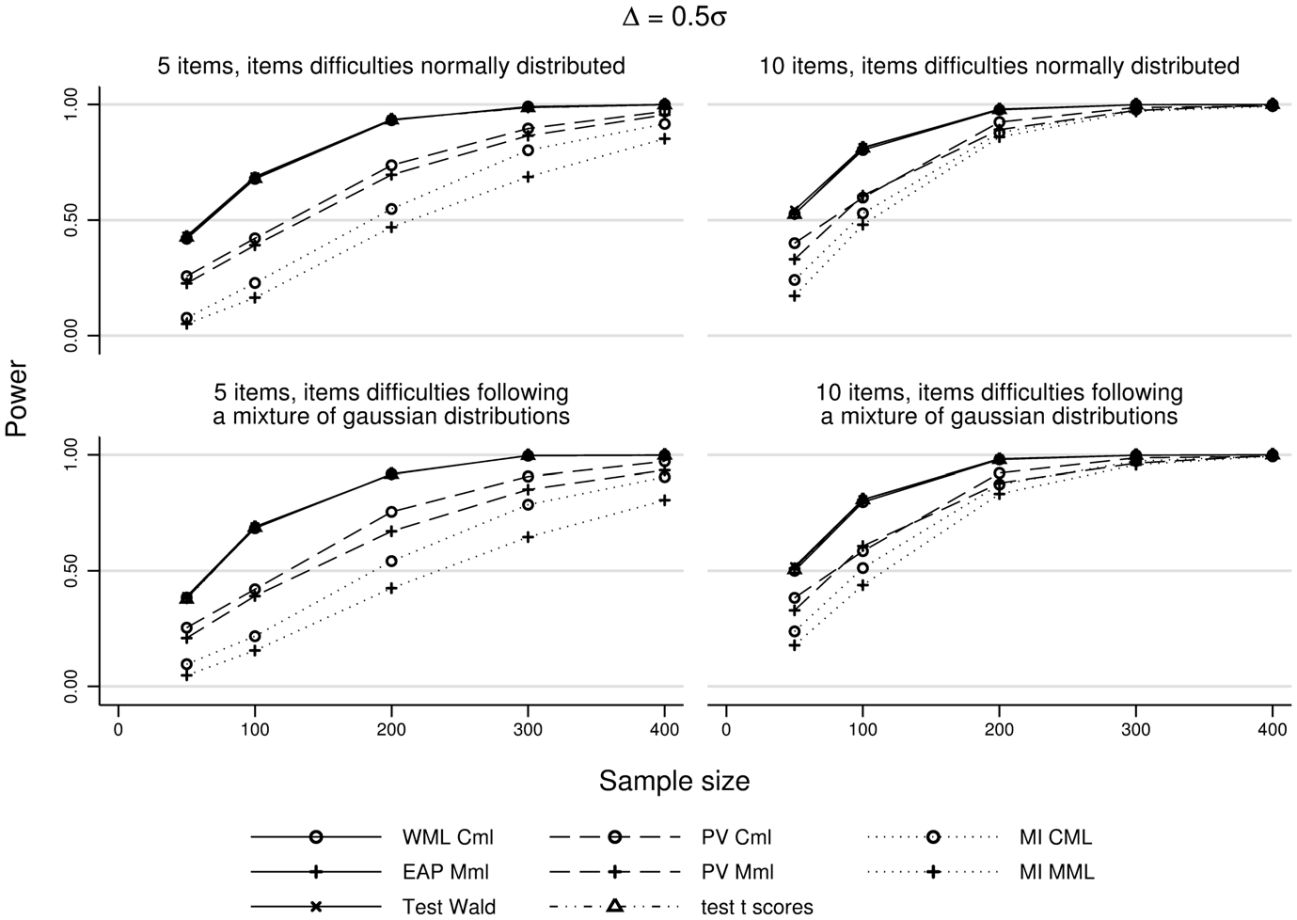
Evolution of the estimated power for the different methodologies controlling the type I error. Evolution of the estimated power depending on the sample size, the items number and the difficulties distribution.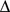 is set at 0.5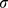 and the items difficulties are considered unknown. CML: conditional maximum likelihood, MML: marginal maximum likelihood, MML-Cov: MML with group covariate, WML: weighted maximum likelihood, EAP: expected a posteriori, PV: plausible values, MI: multiple imputations of PV.

The knowledge of the items difficulties (unknown, well known or imperfectly known) did not affect the comparison methodologies power. We will only present the observed powers for unknown difficulties (table 3 and figure 2).

**Table 3:** Power of the different methodologies for comparing groups on subjective measurements controlling the type I error, for different simulation parameters; the difficulties are considered unknown, the latent traits are normally distributed.

				CTT	IRT						
					CML			MML			MML-Cov
*D_Diff_*	*j*	*n*	Δ	t-test	WML	PV	MI	EAP	PV	MI	Wald
Norm.	5	50	0.2	0.104	0.104	0.078	0.011	0.106	0.056	0.004	0.110
			0.5	0.426	0.419	0.257	0.077	0.427	0.226	0.051	0.431
			0.8	0.801	0.799	0.560	0.336	0.801	0.497	0.256	0.811
		200	0.2	0.306	0.302	0.201	0.044	0.304	0.166	0.023	0.305
			0.5	0.934	0.932	0.737	0.548	0.935	0.696	0.469	0.935
			0.8	1.000	1.000	0.982	0.973	1.000	0.983	0.950	1.000
		400	0.2	0.526	0.519	0.416	0.130	0.523	0.276	0.085	0.523
			0.5	0.999	1.000	0.970	0.916	0.999	0.955	0.852	0.999
			0.8	1.000	1.000	1.000	1.000	1.000	1.000	1.000	1.000
	10	50	0.2	0.116	0.114	0.117	0.033	0.119	0.086	0.018	0.122
			0.5	0.525	0.527	0.400	0.241	0.525	0.330	0.172	0.543
			0.8	0.885	0.883	0.745	0.657	0.888	0.715	0.589	0.892
		200	0.2	0.325	0.328	0.267	0.127	0.325	0.166	0.090	0.330
			0.5	0.978	0.977	0.924	0.878	0.980	0.891	0.859	0.978
			0.8	1.000	1.000	1.000	0.999	1.000	1.000	0.999	1.000
		400	0.2	0.606	0.612	0.514	0.291	0.607	0.422	0.281	0.609
			0.5	1.000	1.000	0.993	0.997	1.000	0.998	0.992	1.000
			0.8	1.000	1.000	1.000	1.000	1.000	1.000	1.000	1.000
Mixt.	5	50	0.2	0.100	0.097	0.086	0.008	0.102	0.063	0.001	0.102
			0.5	0.377	0.384	0.254	0.096	0.381	0.209	0.048	0.389
			0.8	0.772	0.765	0.519	0.305	0.772	0.442	0.200	0.776
		200	0.2	0.263	0.263	0.180	0.041	0.263	0.150	0.025	0.264
			0.5	0.917	0.918	0.755	0.542	0.916	0.671	0.425	0.916
			0.8	0.999	0.999	0.980	0.973	0.999	0.970	0.944	0.999
		400	0.2	0.501	0.494	0.393	0.103	0.499	0.250	0.055	0.500
			0.5	0.999	0.998	0.972	0.905	0.999	0.934	0.805	0.999
			0.8	1.000	1.000	1.000	1.000	1.000	1.000	1.000	1.000
	10	50	0.2	0.101	0.100	0.106	0.025	0.101	0.078	0.013	0.110
			0.5	0.505	0.500	0.383	0.238	0.511	0.329	0.178	0.518
			0.8	0.881	0.884	0.749	0.636	0.883	0.702	0.551	0.888
		200	0.2	0.347	0.347	0.274	0.123	0.352	0.186	0.104	0.356
			0.5	0.980	0.980	0.922	0.873	0.981	0.879	0.832	0.983
			0.8	1.000	1.000	1.000	0.999	1.000	1.000	0.999	1.000
		400	0.2	0.588	0.583	0.490	0.282	0.586	0.388	0.245	0.586
			0.5	1.000	1.000	0.993	0.995	1.000	0.997	0.994	1.000
			0.8	1.000	1.000	1.000	1.000	1.000	1.000	1.000	1.000

CTT: Classical Test Theory, IRT: Item Response Theory, CML: conditional maximum likelihood, MML: marginal maximum likelihood, MML-Cov: MML with group covariate, 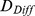: items difficulties distribution, 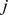: number of items, 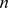: sample size, 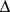: difference between the latent traits means, WML: weighted maximum likelihood, EAP: expected a posteriori, PV: plausible values, MI: multiple imputations of PV, Norm: normal distribution, Mixt: equiprobable mixture of two normal distributions.

The methods respecting the type I error could be grouped into three groups according to their power: (i) the tests with low power, ie the methods based on multiple imputation (MI–MML and MI–CML methods), (ii) the tests with moderate power, ie the methods based on single imputations of plausible values (PV–MML and PV–CML methods), and (iii) the tests with high power, ie the methods based on the comparison of the individual latent traits defined as their average distribution (EAP–MML and WML–CML methods), the Wald-test method and the score comparison t-test.

A global increase of the sample size resulted in an increase of the observed power. In 67% of the cases, this increase was relevant in practice, whatever the values of the other parameters (figure 2). Cases where the difference was not relevant corresponded to observed powers greater than 0.9, resulting in a ceiling effect.

Increasing the number of items resulted in an increase of the observed power. In 55% of the cases, the power increase resulting from the transition from 5 to 10 items was relevant in practice, whatever the values of the other parameters. Cases where this increase was not relevant were either observed powers greater than 0.9, or 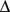 equal to 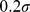.

Finally, the items difficulties distribution did not affect the comparison methods power.

### Bias

#### Position bias

The knowledge of the items difficulties (unknown, well known or imperfectly known) did not affect the position bias estimate (the difference between 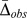 and 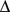). We will only present the estimated position bias for unknown difficulties (table 4).

**Table 4:** Position biases 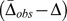 of the different IRT methodologies for comparing groups, for different simulation parameters; the difficulties are considered unknown, the latent traits are normally distributed.

						CTT	IRT										
							CML			MML				MML-Cov			
*D_Diff_*	*j*	*n*	Δ		Δ*_s_*	t-test	WML	PV	MI	EAP	PV	MI	2 steps	EAP	PV	MI	Wald
Norm.	5	50	0		0	0.001	0.001	0.000	0.011	0.001	−0.008	0.001	0.001	0.001	−0.007	0.003	0.001
			0.2		0.193	0.002	0.186	0.183	0.196	**0.102**	**0.094**	**0.102**	0.205	0.205	0.197	0.208	0.205
			0.5		0.483	0.004	**0.463**	**0.460**	**0.473**	**0.263**	**0.256**	**0.264**	0.513	0.511	0.504	0.514	0.511
			0.8		0.769	0.009	**0.744**	**0.743**	**0.754**	**0.438**	**0.431**	**0.439**	**0.823**	**0.821**	0.813	**0.823**	**0.821**
		200	0		0	0.004	0.003	0.006	0.002	0.002	0.002	0.004	0.004	0.004	−0.002	0.005	0.004
			0.2		0.193	0.001	**0.183**	**0.187**	**0.181**	**0.100**	**0.100**	**0.103**	0.202	0.202	0.196	0.204	0.202
			0.5		0.483	−0.002	**0.455**	**0.458**	**0.453**	**0.253**	**0.253**	**0.255**	0.500	0.500	0.494	0.502	0.500
			0.8		0.769	−0.005	**0.725**	**0.728**	**0.723**	**0.423**	**0.423**	**0.426**	0.799	0.798	0.792	0.800	0.798
		400	0		0	0.002	0.002	**0.027**	0.005	0.001	0.000	0.001	0.002	0.002	**0.007**	0.003	0.002
			0.2		0.193	0.006	**0.188**	**0.213**	**0.192**	**0.102**	**0.101**	**0.102**	**0.207**	**0.207**	**0.212**	**0.207**	**0.207**
			0.5		0.483	0.001	**0.457**	**0.482**	**0.461**	**0.254**	**0.253**	**0.254**	0.502	0.502	**0.507**	0.503	0.502
			0.8		0.769	−0.006	**0.723**	**0.747**	**0.726**	**0.420**	**0.419**	**0.420**	0.796	0.795	0.800	0.796	0.795
	10	50	0		0	−0.001	−0.002	0.004	0.005	−0.001	−**0.016**	−0.003	0.000	−0.001	−0.009	−0.005	−0.001
			0.2		0.378	−0.016	0.190	0.196	0.197	**0.128**	**0.112**	**0.126**	0.195	0.194	0.186	0.190	0.194
			0.5		0.944	0.021	0.506	0.511	0.511	**0.344**	**0.329**	**0.343**	**0.517**	**0.517**	0.509	0.512	**0.517**
			0.8		1.504	−0.007	0.790	0.796	0.797	**0.556**	**0.540**	**0.554**	0.807	0.807	0.799	0.803	0.807
		200	0		0	0.005	0.002	**0.009**	0.002	0.002	−**0.013**	**0.008**	0.002	0.003	−**0.010**	0.005	0.002
			0.2		0.378	−0.019	**0.186**	0.194	**0.185**	**0.124**	**0.109**	**0.130**	**0.191**	**0.190**	**0.178**	0.192	**0.190**
			0.5		0.944	0.005	0.493	0.500	0.493	**0.336**	**0.321**	**0.342**	0.504	0.504	0.492	0.506	0.504
			0.8		1.504	0.008	**0.787**	0.794	**0.786**	**0.554**	**0.539**	**0.560**	0.807	0.806	0.794	**0.808**	0.806
		400	0		0	0.004	0.003	**0.014**	0.005	0.001	0.000	0.003	0.002	0.002	−0.004	0.002	0.002
			0.2		0.378	−0.009	**0.192**	0.204	**0.194**	**0.128**	**0.126**	**0.129**	0.196	0.196	**0.189**	0.196	0.196
			0.5		0.944	0.005	**0.493**	0.505	0.495	**0.335**	**0.333**	**0.336**	0.503	0.503	0.496	0.503	0.503
			0.8		1.504	−0.009	**0.778**	**0.791**	**0.780**	**0.545**	**0.543**	**0.547**	0.796	0.796	**0.789**	0.796	0.796
Mixt.	5	50	0		0	0.005	0.005	0.001	0.016	0.001	−0.006	0.002	0.005	0.005	−0.003	0.008	0.005
			0.2		0.181	−0.006	**0.182**	0.178	0.193	**0.093**	**0.085**	**0.094**	0.195	0.195	0.187	0.198	0.195
			0.5		0.451	0.001	**0.472**	**0.467**	0.483	**0.253**	**0.245**	**0.254**	0.510	0.510	0.501	0.512	0.509
			0.8		0.719	−0.013	**0.740**	**0.737**	**0.750**	**0.411**	**0.403**	**0.412**	0.799	0.798	0.790	0.800	0.798
		200	0		0	−0.001	−0.001	0.003	−0.001	−0.001	0.000	0.002	−0.001	−0.001	−0.007	0.001	−0.001
			0.2		0.181	−0.003	**0.184**	**0.188**	**0.182**	**0.094**	**0.095**	**0.097**	0.198	0.197	0.191	0.199	0.197
			0.5		0.451	0.000	**0.467**	**0.470**	**0.465**	**0.247**	**0.247**	**0.249**	0.503	0.502	0.497	0.504	0.502
			0.8		0.719	−0.002	**0.744**	**0.747**	**0.742**	**0.411**	**0.411**	**0.414**	0.801	0.801	0.795	0.803	0.801
		400	0		0	−0.007	−**0.008**	**0.016**	−0.004	−**0.004**	−**0.005**	−**0.004**	−**0.008**	−**0.008**	−0.003	−**0.007**	−**0.008**
			0.2		0.181	0.002	**0.188**	**0.213**	**0.192**	**0.096**	**0.095**	**0.096**	0.203	0.203	**0.208**	0.204	0.203
			0.5		0.451	0.001	**0.467**	**0.492**	**0.470**	**0.246**	**0.245**	**0.246**	0.502	0.502	**0.507**	0.503	0.502
			0.8		0.719	0.003	**0.747**	**0.772**	**0.751**	**0.414**	**0.413**	**0.414**	0.806	0.806	**0.811**	**0.807**	0.806
																	
	10	50	0		0	−0.009	−0.005	0.001	0.001	−0.004	−**0.021**	−0.006	−0.004	−0.005	−0.014	−0.010	−0.004
			0.2		0.353	−0.037	**0.179**	0.186	0.185	**0.115**	**0.100**	**0.113**	**0.181**	**0.181**	**0.172**	**0.177**	**0.181**
			0.5		0.880	−0.004	0.497	0.503	0.504	**0.330**	**0.314**	**0.329**	0.506	0.505	0.497	0.500	0.505
			0.8		1.404	−0.009	0.793	0.800	0.800	**0.543**	**0.527**	**0.541**	0.805	0.805	0.797	0.801	0.805
		200	0		0	−0.008	−0.004	0.003	−0.005	−0.003	−**0.018**	0.003	−0.004	−0.005	−**0.017**	−0.002	−0.004
			0.2		0.353	0.004	0.200	0.208	0.200	**0.130**	**0.115**	**0.136**	0.203	0.203	**0.190**	0.205	0.203
			0.5		0.880	0.004	0.496	0.504	0.495	**0.328**	**0.313**	**0.334**	0.504	0.504	**0.491**	0.506	0.504
			0.8		1.404	−0.013	**0.782**	**0.790**	**0.782**	**0.533**	**0.518**	**0.540**	0.795	0.795	**0.782**	0.797	0.795
		400	0		0	−0.004	−0.002	**0.010**	0.000	−0.001	−0.003	0.000	−0.002	−0.002	−**0.009**	−0.002	−0.002
			0.2		0.353	−0.006	**0.194**	0.206	0.196	**0.126**	**0.124**	**0.127**	0.197	0.197	**0.190**	0.197	0.197
			0.5		0.880	0.003	0.495	**0.508**	0.497	**0.327**	**0.326**	**0.329**	0.503	0.503	0.496	0.503	0.503
			0.8		1.404	0.007	**0.792**	0.803	**0.794**	**0.541**	**0.539**	**0.543**	0.805	0.805	0.798	0.805	0.805

IRT: Item Response Theory, CML: conditional maximum likelihood, MML: marginal maximum likelihood, MML-Cov: MML with group covariate, 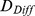: items difficulties distribution, 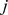: number of items, 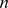: sample size, 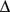: difference between the latent traits means, 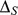: expected difference between the scores means, WML: weighted maximum likelihood, EAP: expected a posteriori, PV: plausible values, MI: multiple imputations of PV, Norm: normal distribution, Mixt: equiprobable mixture of two normal distributions.

Estimated position biases significantly different from 0 appear in bold.

Score t-test, WML-CML, PV-CML, MI-CML, EAP-MML-Cov, PV-MML-Cov, MI-MML-Cov, *2 Steps* and Wald test methodologies did not present any position bias relevant in practice whatever the values of the simulation parameters.

Methods based on a random effects Rasch model without covariates (EAP–MML, PV–MML and MI–MML methods) did not present a relevant position bias when the simulated difference 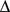 was equal to 0, but presented a position bias systematically relevant in practice when 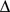 was greater than 0. This bias was then greater than 30% of 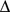 in all the cases.

For methods with a position bias relevant in practice (EAP–MML, PV–MML et MI–MML):

- Neither the difficulties distribution nor the sample size affected the position bias, whatever the values of the other parameters.
- Increasing the items number resulted in a decrease of the position bias relevant in practice: the transition from 5 to 10 items resulted in an average decrease of the position bias of 15% of 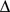, whatever the values of the other parameters.

#### Dispersion biases

The dispersion biases estimates (the difference between 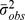 and 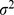) were similar when items difficulties were considered unknown or well known. However, the dispersion biases estimates increased when the items difficulties were considered as imperfectly known: these estimated dispersion biases were greater than those estimated by considering the difficulties as unknown or perfectly known by an average of 15% of 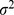, whatever the values of the other parameters. However, the knowledge of the items difficulties did not affect the effect of the other simulation parameters on the observed dispersion biases. We will only present the dispersion biases estimated for unknown difficulties (table 5).

**Table 5:** Dispersion biases 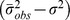 of the different IRT methodologies for comparing groups, for different simulation parameters; the difficulties are considered unknown, the latent traits are normally distributed.

				IRT										
				CML			MML				MML-Cov			
*D_Diff_*	*j*	*n*	Δ	WML	PV	MI	EAP	PV	MI	*2 Steps*	EAP	PV	MI	*Wald*
Norm	5	50	0	**0.768**	**2.067**	**3.646**	−**0.468**	**0.039**	**0.661**	**0.051**	−**0.477**	**0.055**	**0.699**	0.020
			0.2	**0.761**	**2.079**	**3.655**	−**0.463**	**0.055**	**0.676**	**0.052**	−**0.479**	**0.055**	**0.702**	0.019
			0.5	**0.767**	**2.091**	**3.660**	−**0.419**	**0.111**	**0.774**	**0.073**	−**0.463**	**0.084**	**0.736**	**0.043**
			0.8	**0.736**	**2.091**	**3.694**	−**0.392**	**0.172**	**0.853**	**0.051**	−**0.484**	**0.057**	**0.705**	0.019
		200	0	**0.734**	**2.054**	**3.598**	−**0.499**	0.008	**0.614**	**0.014**	−**0.501**	**0.014**	**0.646**	0.007
			0.2	**0.738**	**2.047**	**3.597**	−**0.489**	**0.024**	**0.632**	**0.022**	−**0.497**	**0.023**	**0.656**	**0.014**
			0.5	**0.724**	**2.054**	**3.607**	−**0.471**	**0.054**	**0.680**	0.012	−**0.505**	**0.013**	**0.649**	0.005
			0.8	**0.712**	**2.058**	**3.626**	−**0.425**	**0.134**	**0.790**	**0.017**	−**0.505**	**0.021**	**0.658**	0.010
		400	0	**0.735**	**2.039**	**3.744**	−**0.501**	**0.010**	**0.640**	**0.013**	−**0.502**	**0.013**	**0.600**	**0.009**
			0.2	**0.732**	**2.041**	**3.746**	−**0.497**	**0.016**	**0.647**	**0.011**	−**0.504**	**0.012**	**0.601**	0.007
			0.5	**0.720**	**2.035**	**3.747**	−**0.475**	**0.053**	**0.703**	0.008	−**0.508**	0.007	**0.598**	0.004
			0.8	**0.704**	**2.046**	**3.760**	−**0.432**	**0.124**	**0.806**	**0.009**	−**0.511**	**0.009**	**0.602**	0.005
	10	50	0	**0.540**	**1.184**	**1.915**	−**0.315**	**0.043**	**0.466**	**0.032**	−**0.321**	**0.043**	**0.501**	**0.016**
			0.2	**0.552**	**1.205**	**1.941**	−**0.300**	**0.051**	**0.489**	**0.043**	−**0.311**	**0.054**	**0.517**	**0.028**
			0.5	**0.531**	**1.193**	**1.916**	−**0.295**	**0.064**	**0.505**	**0.021**	−**0.331**	**0.037**	**0.493**	0.007
			0.8	**0.535**	**1.197**	**1.963**	−**0.251**	**0.123**	**0.584**	**0.030**	−**0.327**	**0.039**	**0.506**	0.016
		200	0	**0.508**	**1.140**	**1.848**	−**0.343**	−0.001	**0.438**	0.004	−**0.345**	0.008	**0.420**	0.001
			0.2	**0.503**	**1.150**	**1.829**	−**0.342**	0.002	**0.443**	0.001	−**0.348**	0.006	**0.419**	−0.002
			0.5	**0.511**	**1.164**	**1.872**	−**0.313**	**0.041**	**0.495**	**0.009**	−**0.343**	**0.016**	**0.431**	0.007
			0.8	**0.504**	**1.165**	**1.885**	−**0.278**	**0.089**	**0.563**	0.006	−**0.349**	**0.011**	**0.430**	0.003
		400	0	**0.505**	**1.146**	**1.906**	−**0.344**	0.006	**0.380**	0.004	−**0.345**	0.004	**0.434**	0.003
			0.2	**0.504**	**1.140**	**1.903**	−**0.342**	**0.008**	**0.385**	0.002	−**0.347**	0.000	**0.433**	0.002
			0.5	**0.500**	**1.141**	**1.924**	−**0.321**	**0.035**	**0.423**	0.001	−**0.350**	0.001	**0.432**	0.000
			0.8	**0.496**	**1.159**	**1.940**	−**0.286**	**0.085**	**0.490**	−0.001	−**0.355**	−0.002	**0.433**	−0.002
Bimod	5	50	0	**0.908**	**2.278**	**3.932**	−**0.473**	**0.052**	**0.699**	**0.059**	−**0.483**	**0.068**	**0.734**	**0.032**
			0.2	**0.913**	**2.299**	**3.937**	−**0.466**	**0.066**	**0.713**	**0.064**	−**0.481**	**0.074**	**0.741**	**0.035**
			0.5	**0.905**	**2.289**	**3.934**	−**0.442**	**0.109**	**0.777**	**0.065**	−**0.484**	**0.065**	**0.747**	**0.035**
			0.8	**0.888**	**2.300**	**3.959**	−**0.405**	**0.169**	**0.871**	**0.056**	−**0.492**	**0.055**	**0.739**	**0.027**
		200	0	**0.866**	**2.233**	**3.848**	−**0.514**	0.011	**0.630**	**0.014**	−**0.517**	**0.015**	**0.668**	0.007
			0.2	**0.859**	**2.230**	**3.837**	−**0.515**	0.011	**0.631**	0.008	−**0.522**	0.010	**0.659**	0.000
			0.5	**0.859**	**2.238**	**3.868**	−**0.482**	**0.063**	**0.709**	**0.018**	−**0.516**	**0.021**	**0.672**	0.011
			0.8	**0.845**	**2.242**	**3.873**	−**0.440**	**0.132**	**0.811**	**0.015**	−**0.521**	**0.018**	**0.673**	0.008
		400	0	**0.854**	**2.215**	**3.985**	−**0.524**	−0.002	**0.645**	0.003	−**0.525**	0.004	**0.607**	−0.001
			0.2	**0.856**	**2.224**	**3.988**	−**0.518**	0.007	**0.658**	0.004	−**0.524**	0.004	**0.607**	0.001
			0.5	**0.853**	**2.226**	**4.013**	−**0.488**	**0.058**	**0.730**	**0.012**	−**0.520**	**0.013**	**0.624**	**0.009**
			0.8	**0.843**	**2.236**	**4.029**	−**0.441**	**0.134**	**0.843**	**0.014**	−**0.522**	**0.016**	**0.630**	**0.011**
	10	50	0	**0.580**	**1.237**	**2.000**	−**0.338**	**0.021**	**0.468**	**0.017**	−**0.345**	**0.026**	**0.507**	0.006
			0.2	**0.575**	**1.240**	**1.987**	−**0.337**	**0.032**	**0.471**	0.014	−**0.348**	**0.023**	**0.499**	0.002
			0.5	**0.592**	**1.270**	**2.030**	**−0.304**	**0.080**	**0.529**	**0.027**	**−0.339**	**0.040**	**0.517**	0.014
			0.8	**0.595**	**1.283**	**2.067**	**−0.263**	**0.127**	**0.601**	**0.031**	**−0.340**	**0.043**	**0.527**	**0.017**
		200	0	**0.556**	**1.219**	**1.934**	**−0.355**	0.004	**0.463**	0.007	**−0.357**	**0.014**	**0.441**	0.005
			0.2	**0.556**	**1.214**	**1.939**	**−0.352**	0.005	**0.469**	0.005	**−0.359**	**0.011**	**0.441**	0.003
			0.5	**0.556**	**1.229**	**1.952**	**−0.330**	**0.038**	**0.507**	0.005	**−0.361**	**0.010**	**0.442**	0.002
			0.8	**0.556**	**1.230**	**1.977**	**−0.292**	**0.092**	**0.580**	0.005	**−0.363**	0.008	**0.444**	0.002
		400	0	**0.548**	**1.208**	**1.996**	**−0.363**	**−**0.001	**0.392**	**−**0.001	**−0.364**	**−**0.002	**0.445**	**−**0.002
			0.2	**0.550**	**1.208**	**2.003**	**−0.357**	0.005	**0.402**	0.002	**−0.362**	0.000	**0.448**	0.001
			0.5	**0.554**	**1.231**	**2.017**	**−0.331**	**0.045**	**0.444**	0.005	**−0.361**	0.005	**0.454**	0.004
			0.8	**0.549**	**1.232**	**2.038**	**−0.293**	**0.094**	**0.513**	0.003	**−0.365**	0.002	**0.456**	0.002

IRT: Item Response Theory, CML: conditional maximum likelihood, MML: marginal maximum likelihood, MML-Cov: MML with group covariate, 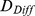: items difficulties distribution, 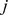: number of items, 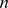: sample size, 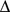: difference between the latent traits means, WML: weighted maximum likelihood, EAP: expected a posteriori, PV: plausible values, MI: multiple imputations of PV, Norm: normal distribution, Mixt: equiprobable mixture of two normal distributions.

Estimated dispersion biases significantly different from 0 appear in bold.

The *2 Steps*, Wald test and PV-MML-Cov methods were the only methodologies which did not present any dispersion bias relevant in practice, whatever the values of the other parameters. The methods for which 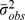 was biased are presented in table 6.

**Table 6:** Dispersion biases of the different methodologies considered for comparing groups on subjective measurements.

Methodologies for comparing subjective measurements	Percentages of the cases where the dispersion bias was greater than 10% of σ^2^	Estimated average dispersion bias
**WML–CML**	**100%**	**+0.669**
**PV–CML**	**100%**	**+1.668**
**MI–CML**	**100%**	**+2.914**
**EAP–MML**	**100%**	**−0.396**
PV–MML	22%	+0.055
**MI–MML**	**100%**	**+0.586**
**EAP–MML-Cov**	**100%**	**−0.427**
PV–MML-Cov	0%	+0.020
**MI–MML-Cov**	**100%**	**+0.559**
*2 steps*	0%	+0.017
*Wald test*	0%	+0.008

CML: conditional maximum likelihood, MML: marginal maximum likelihood, MML-Cov: MML with group covariate, WML: weighted maximum likelihood, EAP: expected a posteriori, PV: plausible values, MI: multiple imputations of PV.

Methodologies with average bias greater than 10% of 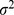 appear in bold.

Increasing the items number resulted in a decrease of the dispersion biases. The transition from 5 to 10 items resulted in a reduction of the dispersion biases by an average of 18%, whatever the values of the other parameters.

The difficulties distribution affected the dispersion biases only for the methods based on a fixed effects Rasch model (WML–CML, PV–CML and MI–CML methods). The transition from a normal distribution to a bimodal Gaussian mixture distribution resulted in an increase of the estimated variance 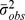 on average of 14%, whatever the values of the other parameters. For the other methods (EAP-MML, PV-MML, MI-MML, EAP-MML-Cov, PV-MML-Cov, and MI-MML-Cov methodologies), the difficulties distribution did not affected the dispersion biases.

Whatever the methods considered, neither the sample size nor the simulated difference 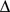 affected the dispersion biases, whatever the values of the other parameters.

### Example

We illustrate the results of this simulation study using data coming from the surveillance program for upper-extremity musculoskeletal disorders (UE-MSDs) in the working population of the French Loire Valley region [28]. One of the objectives of this study was to compare the quality of life of workers according to their occupational category.

In this example, we focused on comparing the physical role level of blue collar workers to that of other workers. The physical role was estimated using the RP (Role Physical) sub-scale of the SF-36 questionnaire [13], including four dichotomous items. We only included individuals aged between 21 and 50 years to take into account the potential effect of age as a confounding variable. 591 blue collar workers and 828 other workers aged from 21 to 50 years completed the SF36 questionnaire. The observed item non-response rate was very low (1.2% in blue collar workers and 1.0% in other workers).

We used all the methods witch did not resulted in an observed type I error significantly greater than 0.05 to compared the physical role according to the workers occupational categories. The methods used were either based on CTT (as the score t-test) or based on IRT (methods based on fixed effect rasch models: WML-CML, PV-CML and MI-CML; methods based on random effect Rasch models: EAP-MML, PV-MML and MI-MML; and methods based on random effect Rasch models including group covariate: the Wald-test method). The score used for the t-test method was calculated as recommended by the SF-36 manual, imputing missing responses by the average observed responses for each individual who responded to at least half of the items [13]. The results of all these comparisons are presented in table 7.

**Table 7:** Measurement of the physical role difference between blue-collar workers and workers from other occupational categories.

	score t-test	CML			MML			Wald-test
		WML-CML	PV-CML	MI-CML	EAP-MML	PV-MML	MI-MML	
difference	**−0.133**	**−0.293**	**−**0.295	**−**0.296	**−0.177**	**−**0.200	**−**0.148	**−0.315**
se	**0.056**	**0.120**	0.167	0.216	**0.073**	0.114	0.135	**0.123**
P-value	**0.017**	**0.015**	0.078	0.183	**0.015**	0.080	0.278	**0.011**

Difference : difference of the physical role measurements between blue-collar workers and workers from other occupational categories according to the comparison methods used.

se : standard error of these differences.

Differences significantly different from 0 appear in bold.

Only four methods highlighted a significant physical role difference according to the occupational category: the Score t-test, the WML-CML, the EAP-MML and the Wald-test methods. These were the methods presenting the highest powers in our simulation study. In this example, their power was substantially identical. Finally, the estimation of the latent trait difference varied according to the different methodologies: EAP-MML and WML-CML provided the lowest estimate of the latent trait difference. We could extrapolate, using the simulation study, that only methods the t-test method and Wald-test were not biased.

In a second step, we randomly generated missing data and compared once again the physical role of blue collar workers to that of other workers to study the effect of missing data on these group comparison methods. The simulated probability of an item non-response was set to 20%. We simulated whether an individual responded or not to an item using Bernoulli trials. Such a method for generating missing data allowed ensuring the non-informativity of missing data. We used the same comparison methods than previously. The results of these comparisons are presented in table 8.

**Table 8:** Measurement of the physical role difference between blue-collar workers and workers from other occupational categories after simulating an item non response rate to 20%.

	score t-test	CML			MML			Wald-test
		WML-CML	PV-CML	MI-CML	EAP-MML	PV-MML	MI-MML	
difference	−0.107	−0.296	−0.212	−0.230	−**0.170**	−0.199	−0.144	−**0.321**
se	0.065	0.151	0.169	0.237	**0.081**	0.118	0.194	**0.128**
P-value	0.104	0.050	0.210	0.347	**0.036**	0.093	0.476	**0.012**

Difference : difference of the physical role measurements between blue-collar workers and workers from other occupational categories according to the comparison methods used.

se : standard error of these differences.

Differences significantly different from 0 appear in bold.

The estimation of the score difference between groups using the t-test method varied from more than 20% depending on whether data was complete or missing. Although missing data were fully non-informative, the estimation of the score difference between groups was lower in case of missing data. On the other hand, the estimation of the latent trait difference between groups using non-stochastic IRT methods (WML-CML, EAP-MML and Wald-test methods) did not seem impacted by the presence or absence of missing data: for these considered methods, the latent trait difference estimation varied from less than 5%. Finally, when data was missing, only two methods highlighted a significant physical role difference according to the occupational category: the EAP-MML and the Wald-test methods. The Score t-test method no longer highlighted such a difference.

## Discussion

### Choice of the Most Efficient Methods for Comparing Two Groups of Individuals on PRO Data

The preferred methods of comparison are those for which the type I error is not significantly greater than 5%. Those with the greatest power will then be preferred. Among them, those with the most reduced biases will be the ones to consider.

#### Type I error

The methods based on the individual latent traits analysis estimated by a Rasch model with group covariate (EAP–MML-Cov, PV–MML-Cov and MI–MML-Cov method) and the *2 Steps* method resulted in an unacceptable rate of type I error. These methods were therefore unsuitable for latent traits comparison.

#### Power

Among the methods controlling the type I error, methods based on multiple imputations of plausible values (MI–CML and MI–MML methods) had the lowest power. This power loss can be associated with their dispersion biases. Their estimated variances 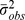 were all biased and greater than the simulated variances 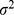. These biases were related to the addition of the within-subject variance component to the latent traits variance estimate. This within-subject variance illustrates in fact the imprecision related to the individual latent traits estimate, and is not related to the individual latent trait variability [20]. Indeed, in the framework of cross-sectional studies, each individual latent trait is measured only once, which does not make it possible to assess individual latent traits variability. Therefore, if one focuses on the latent trait dispersion parameters within a population at a given time (as in cross-sectional studies), only the between-subject variance should be taken into account.

Methods based on plausible values (PV–CML and PV–MML methods) presented a moderate power. For the PV–CML method, this limited power can be linked with the increase of the dispersion biases associated with the use of plausible values. Methods based on conditional likelihood for estimating individual latent traits are known to result in a biased and increased variance estimate [29]. The addition of a between-subject variance component with plausible values methodologies can only increase this bias. For the PV–MML method, this limited power can be linked with the dispersion biases due to the use of Bayesian expected a posteriori estimates for estimating individual latent traits [30]. These expected a posteriori estimates are indeed shrunk to their a priori value. Thus, the 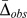 are decreased compared to the simulated 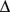.

The following methods WML–CML, EAP–MML, Wald-test and score t-test presented the highest powers. These methods’ powers were almost identical.

#### Biases

As expected, the WML–CML method did not lead to any relevant position biases in practice but to dispersion biases when estimating the latent traits distribution parameters [31]. The estimated variance 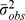 was indeed greater than the simulated variance 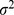. The EAP–MML method leaded to position and dispersion biases when estimating the latent traits distribution parameters. The 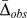 were minimized compared to the simulated 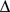, as well as the estimated variance 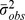 that was less than the simulated 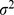. These biases were related to the shrinkage phenomenon associated with the Bayesian posterior estimates of the individual latent traits [29].

The Wald-test and the scores t-test methods did not lead to any position nor dispersion bias when estimating the parameters of the latent traits distribution.

#### Influence of the simulation parameters

For all the considered methods, an increase in the sample size involved an increase of the tests’ power. However, no link has been found between the sample size and the magnitude of the observed biases.

An increase in the number of items involved a reduction of the position and dispersion biases, and an increase of the tests’ power. This phenomenon is known [32], and some authors recommend to estimate the variances and averages of latent traits by a Rasch model only if the questionnaire comprise a minimum of 10 items [17]. The Wald-test method providing unbiased estimates even with less than 10 items, this recommendation should not necessarily be followed to perform group comparisons using this method. The power rise due to the number of items increase is due to the subjective nature of the latent traits. Latent variables being not directly observable, their estimate accuracy is largely dependent on the tool used to perform these estimates. Increasing the items number of a questionnaire leads to an increase of the accuracy of the latent traits estimation, and thus to an increase of the tests’ power performed with this questionnaire [33].

Finally, a change in the distribution of the item difficulties did not affect the tests’ power, nor their position biases. However, such a change in the item difficulties distribution involved a variation of the dispersion biases for methods based on a Rasch model, and a variation of the scores variance for methods based on the score analysis. In addition, a ceiling effect was observed when the items distribution resulted from a mixture of Gaussian distributions.

#### Influence of the knowledge on the items difficulties

Several scenarios were considered, the difficulty parameters of items being considered as unknown, well known or imperfectly known.

The parameters chosen to simulate imperfectly known difficulties corresponded to a rather poor precision that might be rarely encountered in real situations. However, the impact of the knowledge on the items difficulties remained negligible on the power estimate of the different comparison methods, as well as on the estimated position biases [33]. Only the variance estimate of the latent traits was slightly increased when the items difficulties were imperfectly known.

It is therefore possible to use difficulty parameters previously estimated during an IRT based questionnaire validation to perform group comparisons with IRT-based methods on PRO measurement in clinical trials or epidemiological studies. Moreover, choosing these difficulty parameters allows comparing patients coming from different studies that made use of the same questionnaire.

#### Influence of missing data and limitations of the study

A limitation of this study is that it does not take into account the possible presence of missing data. An illustrative real data example has been used for this purpose. This example illustrates some very important changes in the properties of the considered comparison methods according to whether data is missing or not. Even if missing data is not informative, which is the most favourable case, the CTT based method seems to be very disturbed by such missing data. On the contrary, the IRT-based methods seem less affected by the presence of missing data, in view of the example presented in this article. These differences can be explained by the fact that with IRT, an individual latent trait is directly estimated by analysing the items the individuals have answered, without taking account of the missing item answers. With Rasch family models, such estimations are consistent because of the specific objectivity property of such models. On the other hand and with the CTT, the measurements are performed by calculating scores. When data is missing, the score calculation is only possible by performing missing data imputations, which potentially generates biases. It seems important to continue this study by comparing these different group comparison methods in case of missing data considering different scenarios of missing data process, leading to informative or non-informative missing data (missing completely or not at random).

Even though more and more questionnaires are validated by IRT methods, Rasch models investigated in this study may seem too restrictive to be applied to all the situations of clinical research studies (in this study, the items were necessarily dichotomous, and the items difficulties should be independent of the patients groups studied). It appears necessary to pursue this study by analysing extensions of the Rasch model, allowing for polytomous items analysis (as the Partial Credit Model or the Rating Scale Model), and the analysis of items with difficulties that are dependent of the patients groups studied (by integration of the differential item functioning phenomenon in the studied models).

### Conclusion

If data follow both a Rasch model and a CTT-based model, the most appropriate methods to compare two groups of patients on PRO measurements are the scores comparison by t-test when analysing such variables with CTT, and the covariate Wald test, performed with a random effect Rasch model including a group covariate, when analysing such variables with IRT. These two methods displayed very similar powers and unbiased estimates.
